# Sparing Is Caring: Hormonal Retreatment in Women with Recurrent Endometrial Cancer after Fertility Preservation Management—A Single Centre Retrospective Study

**DOI:** 10.3390/healthcare11071058

**Published:** 2023-04-06

**Authors:** Ida Pino, Maria Di Giminiani, Davide Radice, Ailyn Mariela Vidal Urbinati, Anna Daniela Iacobone, Maria Elena Guerrieri, Eleonora Petra Preti, Silvia Martella, Dorella Franchi

**Affiliations:** 1 Preventive Gynecology Unit, European Institute of Oncology IRCCS, Via Ripamonti 435, 20141 Milan, Italy; 2 Unit of Obstetrics and Gynecology, ASST Fatebenefratelli-Sacco, Department of Biological and Clinical Sciences L. Sacco, University of Milan, 20157 Milan, Italy; 3 Division of Epidemiology and Biostatistics, IEO European Institute of Oncology IRCCS, Via Ripamonti 435, 20141 Milan, Italy; 4 Department of Biomedical Sciences, University of Sassari, 07100 Sassari, Italy

**Keywords:** endometrial cancer, fertility-sparing treatment, recurrence, retreatment

## Abstract

Fertility-sparing treatment (FTS) of endometrial cancer (EC) has a high rate of remission but also a high rate of relapse (10–88%). Many women still wish to conceive at the time of relapse, but results regarding retreatment are still lacking. This study aims to evaluate the safety, oncological and pregnancy outcomes of repeated FST in women with recurrent EC. This is a retrospective single-center study that recruited patients who had uterine recurrence after achieving a complete response (CR) with FST for FIGO stage IA, well-differentiated (G1), endometrioid EC. All eligible women underwent a second FST. Among 26 patients with recurrence, 6 decided to receive a hysterectomy and 20 received fertility-sparing retreatment. In total, 17 out of 20 women (85%) achieved a CR in a median time of 6 months. A total of 2/20 women showed a stable disease and continued the treatment for a further 6 months and finally achieved a CR. In total, 1/20 women showed disease progression and underwent demolitive surgery. After relapse and a CR, 14 patients attempted to become pregnant, among whom 7 became pregnant (pregnancy rate 50%—life birth rate 29%). Secondary FST is a safe and effective option for women who desire to preserve fertility after the recurrence of early-stage EC.

## 1. Introduction

Endometrial cancer (EC) is the most common gynecologic tumor in developed countries and the fifth most common cause of cancer in women worldwide [1,2]. Although typically considered as a postmenopausal cancer, between 15% and 25% of cases occur in premenopausal women, 3–5% of whom are younger than 40 years [3].

Due to the delay in the first pregnancy of modern-day women, more than 70% of patients aged under 40 years are nulliparous at the time of diagnosis; therefore, increasing efforts are needed to preserve fertility [4].

Risk factors for EC in young age include conditions characterized by prolonged estrogen exposure not opposed by progesterone, such as polycystic ovary syndrome (PCOS), obesity, infertility and nulliparity [5].

PCOS is commonly diagnosed in women under 35 years of age with EC. The development of EC in these patients is not only attributed to chronic anovulation but also to insulin resistance. In fact, they usually present a concurrent diagnosis of insulin resistance and obesity as well as a more advanced stage of the disease [6]. Comorbidities such as diabetes, obesity, nulliparity and infertility are all independent risk factors for EC [7].

Hyperinsulinemia and insulin resistance have also been related to a higher risk of EC in many studies [8].

Insulin exhibits mitogenic and antiapoptotic properties and shares downstream signaling pathways with insulin-like growth factor-1, which contributes to endometrial proliferation [8].

According to the European Society of Gynecological Oncology (ESGO)/European Society of Human Reproduction and Embryology (ESHRE)/European Society for Gynecological Endoscopy (ESGE) guidelines, these criteria should be considered before starting a FST of EC: G1 endometrioid EC; disease limited to the endometrium on Magnetic Resonance Imaging (MRI) or transvaginal ultrasound (TVUS); absence of metastatic disease on imaging [9].

In this recent guideline, the standard treatment protocols for the conservative management of EC are summarized. They are based mainly on progestins, given both orally (Megestrol Acetate (MA) or Medroxyprogesterone acetate) and/or via a Levonorgestrel Intrauterine Device (LNG-IUD). Alternative approaches include the administration of aromatase inhibitors and/or a GnRH analogue (GnRHa) [9].

New therapeutic options are currently under investigation, and among them, Metformin (MET) is one of the most interesting [10,11].

According to current evidence, although not confirmed by all studies, adding MET to progestin treatments increases both the response rate and relapse-free survival [12,13].

Overall, the fertility-sparing management of EC showed good CR rates and excellent overall survival. However, 9–40% of patients develop recurrence after FST, with a median time to recurrence of 20 months (range 3–357) [14,15,16,17,18,19].

Several women still wish to preserve fertility at the time of relapse, and 63–73% of these patients prefer a fertility-preserving retreatment over radical surgery [20,21].

According to the ESGO/ESHRE/ESGE guidelines, FTS can be considered for intrauterine recurrences only in highly selected cases under strict surveillance (Level of evidence IV, Grade B) [9].

Despite the strong clinical interest, universal results regarding the oncological and obstetrical outcomes of hormone retreatment in patients with EC recurrence are still lacking.

The aim of this study is to evaluate oncological and pregnancy outcomes of repeated FST in women with recurrent EC after achieving a complete response with FST.

## 2. Materials and Methods

Patients who developed intrauterine recurrence after achieving a CR to an initial FST for early-stage EC were selected for a retrospective analysis.

This study was conducted at the Preventive Gynecology Unit of the European Institute of Oncology, Milan, Italy, from 2005 to 2022, and was approved by our Institutional Review Board. Informed consent was obtained from all the women involved.

The inclusion criteria were: (1) age between 18 and 43 years; (2) strong desire for pregnancy; (3) recurrence after achieving a CR with FST in stage IA according to Federation of Gynecology and Obstetrics (FIGO) classification, G1 endometrioid EC; and (4) recurrence confined to the endometrium without myometrial invasion.

Exclusion criteria were: (1) no desire to preserve fertility; (2) myometrial invasion on imaging; (3) diagnosis of moderately (G2) or poorly differentiated (G3) EC; or (4) metastasis.

All patients underwent a pretreatment evaluation that included (1) counselling about fertility options and the nonstandard nature of FST for recurrent disease; (2) TVUS or MRI to exclude myometrial invasion, synchronous ovarian cancer or extrauterine disease; and (3) a pathological review of original slides if diagnosis was made at different institutions.

Recurrence was defined as atypical endometrial hyperplasia (AEH) or endometrial cancer on follow-up endometrial biopsy.

Dedicated gynecological pathologists conducted all histological diagnoses.

The treatment protocols used in the secondary FST varied over time. They were based on the previous therapy performed on each patient and the type of recurrence (AEH or EC).

The treatment protocol included the following hormonal therapies:

- GnRHa + LNG-IUD;
- MA 160 mg/day + LNG-IUD;
- MA 160 mg/day + LNG-IUD + MET 500 mg 3 times per day;
- Continuous oral progestin alone (MA 160 mg, Desogestrel 75 mcg).

The GnRHa used was Triptorelin Acetate in a monthly depot injection of 3.75 mg, and the LNG-IUD was Mirena^®^ (Bayer Health Care Pharmaceutical Inc., Wayne, NY, USA).

The response to treatment was evaluated on endometrial biopsies collected via an office hysteroscopy after 3–6 months of therapy.

The final response was classified as a CR if the final histological examination showed a normal endometrium; partial response (PR) when AEH was diagnosed in patients with initial EC; stable disease (SD) in case the of the persistence of the same histological diagnosis; and progression of disease (PD) when women with initial G1 EC developed G2 or G3 EC.

According to our protocol, women who reached a CR were allowed to seek pregnancy.

Medical reports were used to collect data on the demographic and clinical characteristics of patients, as well as their fertility outcomes.

The patients’ characteristics that were categorized as continuous and categorical variables were summarized by means and Standard Deviation (SD) and count and percent, respectively. Between-group comparisons were tested for significance using the two-sample Wilcoxon test (continuous variables) and the Fisher’s exact test (categorical variables). The median time to complete a response was estimated by using the Kaplan–Meier (KM) method and was plotted as a cumulative incidence function (1-KM). All tests were two tailed and considered significant at the 5% level. All the analyses were conducted by using SAS 9.4 (Cary, NC, USA).

## 3. Results

We identified 80 patients who satisfied the inclusion criteria for FST and tried primary conservative management due to stage IA, G1, endometrioid EC from 2005 to 2022. Of them, 61 patients achieved a CR after initial FST. Among these 61, 26 patients (42.6%) had a recurrence and were selected for this retrospective analysis.

Regarding the pathology at recurrence, 17 patients (65%) were diagnosed with AEH and 9 (35%) with endometrioid EC (8/9 G1 and 1/9 G3).

Six women (23%), including one patient with AEH and five patients with endometrioid EC, underwent demolitive surgery, including a hysterectomy, at the time of recurrence.

In these cases, the histological uterine specimens after hysterectomy showed FIGO IA stage EC or AEH in four women and was negative in two cases, respectively.

**Figure 1:**
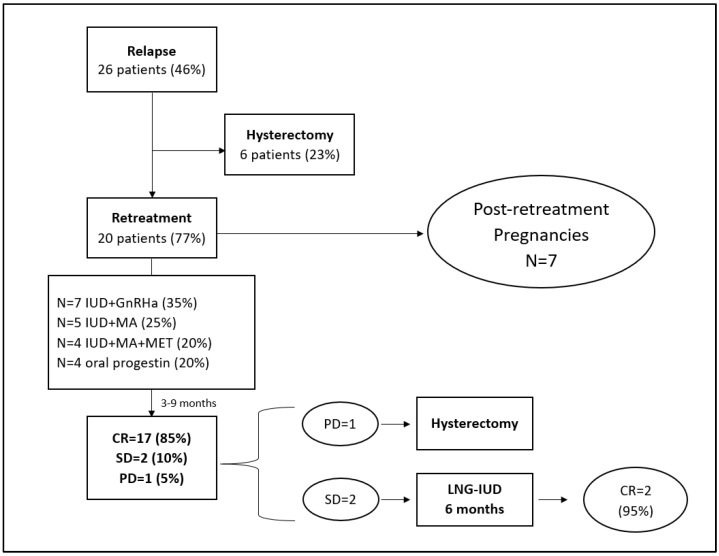
Flow chart of oncological and obstetric outcomes after retreatment.

The remaining 20 patients (77%) underwent a secondary fertility-sparing treatment. The outcomes are shown in Figure 1.

Table 1 provides an overview of the principal characteristics of the study population.

There were no statistically significant differences between the women who received the first FST and the women selected for the secondary conservative treatment, except for the age at menarche and the duration of the follow-up, which was significantly longer in the patients who underwent retreatment.

The maintenance therapies after CR were used at the time of recurrence, the medical treatment chosen for the retreatment and the ovarian stimulation performed before the relapse are reported in Table 2.

**Figure 2:**
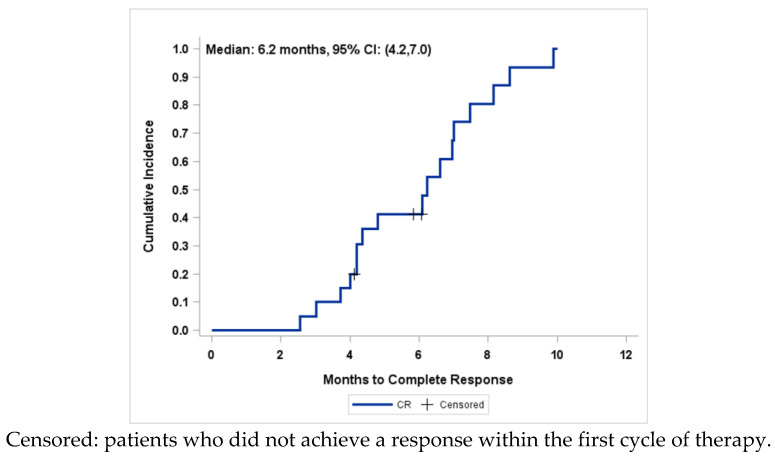
Cumulative incidence of complete responses, N = 20 patients.

The retreatment was performed for a median time of 6 months (range: 3–9 months). A CR was achieved in 17/20 patients (85%) (Figure 2).

Of the remaining patients, 2/20 (10%) showed SD on the follow-up biopsy and continued the treatment with the LNG-IUD for 6 months more. After that, both patients achieved a CR (95% CR at 15 months). Only one patient (5%) showed PD at the follow-up biopsy (endometrioid endometrial carcinoma G3) and underwent demolitive treatment. However, the final histological uterine specimens after hysterectomy showed AEH.

Three out of nineteen (15.8%) patients developed a second relapse after a median time of 24 months (range 12–33), including two with AEH and one with EC. All the patients were receiving cyclic progesterone therapy as they were seeking to conceive, whereby two were seeking to conceive through assisted reproductive technologies (ART). The two patients diagnosed with AEH showed a strong desire to further preserve their fertility and were treated again conservatively with an LNG-IUD and MA. Both achieved a complete response at the 6-month follow-up biopsy (CR = 100%). The median follow-up after relapse was 41 months (range 4–130). Of the 20 retreated patients, only 7 underwent hysterectomy: 2 for endometrial carcinoma, 1 for a metachronous ovarian tumor and 4 for completed childbearing. The remaining women were still in follow-up and were maintaining a CR. All the patients who tried retreatment are alive without evidence of disease.

Among the 20 patients receiving secondary FST, 14 patients (70%) attempted to become pregnant after relapse, 11 (79%) through ART, which resulted in a total of 7 pregnancies and a pregnancy rate of 50%. Among them, only 4 out of 14 patients gave birth to a live baby, which resulted in a live birth rate of 29%; the miscarriage rate was 42.9% (Table 3).

## 4. Discussion

In our study, the CR rate after secondary FST for patients with an intrauterine recurrence of EC after primary FST was 95% after 15 months of treatment, with an 85% CR rate observed at 6 months.

A considerable pregnancy rate (50%) was obtained in the patients but with an unsatisfactory live birth rate (29%). During the subsequent follow-up period, only three patients (16%) had a second recurrence, two of which were successfully retreated with hormone therapy. All patients who received retreatment are alive without evidence of disease. Therefore, our study supports the safety and the efficacy of secondary FST in women with EC recurrence who still wish to preserve fertility.

Our findings are in line with the recommendations of the ESGO/ESHRE/ESGE guidelines, which allow repeated FST for women who experience intrauterine recurrence after an initial successful FST for EC [9].

Fertility-sparing management is well known and has been widely used in young women with EC who wish to conserve fertility. Prior research has demonstrated that conservative treatment has a high rate of remission, but also a high rate of relapse, ranging from 9 to 40%; this represents the biggest concern with this management [14,15,16,17,18,19].

There is no consensus on the management of EC recurrence after fertility preservation as most patients undergo definitive surgical treatment including hysterectomy. Anyway, some women still wish to conceive after recurrence. Limited studies have evaluated the oncological outcomes of repeated FST in patients with relapse after a primary FST. In the literature, the CR rate with secondary FST ranges from 76 to 98% [21,22,23,24,25].

In our study, the complete response rate after secondary FST was 95%, which is consistent with the results of previous studies.

In our study, similar to others, it was found that recurrences following primary FST are predominantly restricted to the endometrium and have a good overall prognosis.

This contrasts with the poor prognosis observed in patients with EC recurrence after standard surgical treatment [26].

This is likely because patients undergoing FST are carefully selected based on specific criteria, such as having low-grade endometrioid cancer confined to the endometrium, which is associated with a very low risk of metastasis [27].

The challenge in establishing clear boundaries for FST may be related to multiple factors that impact its efficacy. The most crucial variables include the tumor’s clinicopathological characteristics (such as histological type, grade and the presence of myometrial invasion or LVSI), the type, dose, duration of therapy performed and the follow-up schedule [28].

All the relapses in our study were intrauterine recurrences of G1 endometrioid EC without myometrial invasion.

In the absence of myometrial infiltration, the risk of pelvic and/or para-aortic lymph node involvement for G1 EC without myometrial invasion is lower than 1% [27]. The grade is one of the most important predictors of the response to hormonal treatment. The response rate to FST with Medroxyprogesterone acetate decrease from 37% in women with G1 EC to 9% in women with G3 disease [29].

Currently, there is limited evidence supporting the safety of FST in patients with G2 EC, and there is minimal experience with retreatment in this patient subgroup.

Falcone et al. analyzed 23 women with intramucosal, G2 endometrioid EC with an overall CR rate of 73.9%. All the patients recurred, and among them, one patient refused a hysterectomy after relapse and was retreated with a combined progestin therapy (LNG-IUD + MA); the CR was archived within 6 months [30].

As previously reported, our study excluded patients diagnosed with moderately or poorly differentiated EC; anyway, it is another limitation of FST that requires further investigation.

Although myometrial infiltration is considered an exclusion criterion for FST, recent research suggests that women with minimally infiltrating G1 EC may be suitable candidates for conservative management [31].

Among the demographic parameters analyzed, only the age of the menarche was found to be significantly lower in patients with recurrence undergoing retreatment compared to those who achieved CR at the first FST and did not develop an EC recurrence. This could be attributed to a longer exposure to estrogen and anovulatory cycles.

The rate of PCOS was higher in our cohort (15%) than that reported in the general population (5–8%); this confirms the three-fold increased risk of EC in women with PCOS previously showed in the literature [32].

PCOS is commonly linked with chronic anovulation and infertility, which may lead to a worsening of fertility outcomes and could potentially contribute to our low birth rate [33].

Furthermore, women diagnosed with both PCOS and EC have been found to respond less to treatment with Medroxyprogesterone acetate [6].

On the other hand, the polycystic ovarian morphology, which can be determined with TVUS, might be a good prognostic factor in patients with EC who achieved a CR after FST with progestin, regardless of their BMI [7].

Considering that women diagnosed with PCOS are less likely to undergo FST due to their higher frequency of advanced disease and failure of progestin therapy, greater attention should be given to this condition [6].

An early diagnosis and the appropriate treatment of this syndrome are important for the prevention of EC in young woman.

Moreover, it is crucial that women with PCOS are aware of the positive effects of lifestyle changes and medical treatment to reduce their risk of EC as well as their metabolic syndrome.

It is very important considering that two patients in our series were already receiving treatment with antihypertensive or diabetes medications at the time of EC diagnosis, despite their young age.

Additionally, we observed a high prevalence of endometriosis (18%) and infertility (28%) in our study population. This could be partially attributed to the fact that many patients were diagnosed with EC during diagnostic investigations conducted for infertility. Nevertheless, both conditions have previously been associated with an increased risk of developing EC [34,35].

FST is not curative; hence, further attention should be paid to the importance of maintenance therapy if women do not attempt pregnancy immediately after the CR.

The use of maintenance therapy after achieving CR was significantly associated with improved recurrence-free survival, and it might be useful to decrease the recurrence after a successful FST [24].

Although there was no significant difference between the groups according to the type of maintenance treatment, due to the small sample size, the patients receiving continuous progestin or IUD + MET showed the lowest number of relapses, as shown in Table 2. Even if these results need to be confirmed, the combination of IUD and MET could reduce the recurrence rate during maintenance therapy, as previously described [12].

As in our study, progestins combined with MET exhibited an additional benefit in reducing the recurrence rate, particularly in overweight women (BMI ≥ 25 kg/m^2^) but also in patients with a normal weight [13,36].

Young patients with EC frequently have a history of obesity, which is usually associated with prolonged and unopposed estrogen exposure, which accounts for the increased risk of EC in obese women [37,38].

The high BMI of our study population (mean: 24.4 kg/m^2^) could have played a role in the improved efficacy of the maintenance regimen containing MET.

Furthermore, none of the patients with relapse were receiving treatment with GnRHa.

The therapeutic regimens used in the secondary FST were individualized based on each patient’s previous therapy, the type of recurrence (AEH or EC) and the best evidence available at the time of relapse. There is still no consensus in the literature on secondary conservative treatment after the recurrence of EC.

In our Institute, the protocols for the FST of EC have changed over the years, but combined therapeutic regimens have always been preferred. This approach was developed to maximize the effectiveness of the therapy through different medical treatments or higher doses of progestin and possibly reduce the side effects.

The first therapeutic approach consisted of GnRHa + LNG-IUD. However, few studies have confirmed the efficacy of this association [39,40,41].

Medroxyprogesterone acetate and MA are the most frequently used oral progestins in FST protocols [42].

Even if there is no agreement in the literature, MA showed a higher remission rate than Medroxyprogesterone acetate [40,43].

A recent systematic review suggests a higher efficacy of a high-dose progestins protocol compared to the low-dose one [44].

Nevertheless, administering progestins orally at high doses for a prolonged period can lead to side effects. However, they can be partially reduced through intrauterine administration. An LNG-IUD has been shown to be effective alone in FST in most cases of early stage EC [45,46].

Several studies have shown that the response rate is higher with combined oral progestin/an LNG-IUD than with single treatment during the initial FTS [47,48].

Because of this evidence, another therapeutic regimen, based on the combined use of LNG-IUD + MA, was introduced into our treatment protocol.

MET has the potential to inhibit tumor growth by modulating glucose metabolism. Additionally, it may increase the efficacy of progestin treatment by upregulating the expression of progesterone receptors in EC [49].

Furthermore, the reduction in body weight that is often induced by MET treatment could have a beneficial effect during FST in terms of both oncological and reproductive outcomes [50].

Since many encouraging data have been published on the use of MET in the FST of EC and the tolerability of its side effects, MET was added to our protocol in 2017.

The role of MET in the FST of EC and AEH are planned to be examined in a prospective randomized study [51].

The CR rate for retreatment was so high that significant differences could not be found between the various therapeutic regimens for secondary FST in our small study.

Usually, the secondary treatment regimens include high-dose oral progestin. Anyway, other methods such as GnRHa-combined therapy has an advantage on weight control compared with progestin therapy. The latter usually avoids the weight gain frequently associated with high-dose oral progestin. Furthermore, a GnRHa-combined regimen could be an alternative option for women with recurrent disease that are unable to take progestin, e.g., due to abnormal liver function [52].

In our study, 80% (16/20) of the patients received combined therapy with an IUD for secondary FTS depending on the treatment protocol at the time of relapse and individual patient conditions. However, concurrent use of an LNG-IUD was not significantly associated with a CR to secondary FST, realistically because of the small sample size.

Future randomized trials are necessary to confirm the role of the LNG-IUD in the management of repeated FST.

The use of ART has not been shown to increase the risk of EC recurrence or worsen oncological outcomes, as demonstrated by several authors [53,54].

In our study, 11/14 (79%) patients used ART prior to the EC recurrence, including 5/11 with ovarian stimulation. This may reflect the interruption of the maintenance therapy more than a risk factor associated with the regimen used.

It is not uncommon for young EC patients to have synchronous or metachronous ovarian cancer. Previous studies reported a prevalence of these tumors of 5–29% [3].

In this study, we observed only one case of metachronous endometrioid ovarian cancer stage IA G2, but this patient was undergoing a conservative management of both ovarian and endometrial endometrioid carcinoma. Therefore, when intrauterine recurrence is identified, it is crucial to verify that the tumor is confined to the endometrium, and a complete evaluation is essential to ensure that the woman meets the eligibility criteria for retreatment. Furthermore, an adequate imaging follow up is needed since the ovaries are conserved.

In our Institute, we routinely perform a transvaginal ultrasound every 6 months and after each hysteroscopy in women undergoing FST.

Our study population showed a good pregnancy rate (50%) despite a greater prevalence of infertility before EC diagnosis (30%) compared to the general population (10–15%) [55]. In our series, none of the patients experienced pregnancy prior to the diagnosis of EC.

The live birth rate was 29%, which was lower than that observed in the reference population.

Considering that 20 out of 26 women (77%) with EC recurrence still desired pregnancy and chose a secondary FST, despite a thorough counseling on the oncological risks and the limited data supporting retreatment, even a low live birth rate can be acceptable.

The rate of spontaneous abortions was notably higher in our study (43%) than that reported in healthy populations (15–20%) [56].

The results are less worrisome considering that 11 women resorted to ART and the miscarriage rate in this subgroup, which took into account preclinical pregnancy loss in the general population, increased to 30% [57].

Moreover, our population showed a high prevalence of endometriosis and infertility, which maybe affected pregnancy outcomes.

The present study is characterized by rigorous patient selection, an accurate methodology, a comparison of different combined hormonal therapeutic regimens and a long follow-up period, which are significant strengths. Nevertheless, this study has some limitations, such as the retrospective single-center design and the relatively small sample size.

Furthermore, the treatment of these patients was not randomized, and heterogeneous patients received a variety of oral and intrauterine combinations. Lastly, we did not evaluate the molecular classifications and the expression of estrogen and progesterone receptors [58,59,60,61].

In the future, it is important to assess the feasibility and the safety of higher-lines FST for patients experiencing second or subsequent EC recurrences. Additionally, research efforts should aim to identify more effective treatment approaches for nonresponders by utilizing molecular classification and predictive biomarkers.

## Figures and Tables

**Table 1:** Patients’ characteristics: summary statistics of continuous and categorical variables ^a^.

			Group		
Characteristic		All patients N = 61	FS-Treatment N = 41	FS-Retreatment N = 20	*p*-Value ^b^
**Age at diagnosis, years**		34.3 (5.6)	34.7 (5.6)	33.5 (5.2)	0.36
**Age at menarche, years, N = 54 ^c^**		12.0 (1.6)	12.4 (1.7)	11.4 (1.1)	**0.02**
**Body Mass Index, N = 59 ^d^**		24.6 (5.9)	24.6 (6.1)	24.4 (5.4)	0.88
**Follow-up, months**		54	45	72	**<0.01**
**PCOS**		10 (16.4)	7 (17.1)	3 (15.0)	1.00
**Hypertension**		3 (4.9)	2 (4.9)	1 (5.0)	1.00
**Diabetes**		1 (1.6)	0	1 (5.0)	0.33
**Endometriosis**		11 (18.3)	8 (20.0)	3 (15.0)	0.74
**Infertility**		17 (27.9)	11 (26.8)	6 (30.0)	1.00
**Ovarian cancer**		10 (16.4)	6 (14.6)	4 (20.0)	0.72
**Smoke**	Never	42 (79.3)	26 (76.5)	16 (84.2)	
	Former	3 (5.7)	3 (8.8)	0	
	Present	8 (15.1)	5 (14.7)	3 (15.8)	0.65

^a^ Mean (SD), median for follow-up; SD = Standard Deviation; FS = fertility sparing; ^b^ *t*-test for independent variables or Wilcoxon test; ^c^ FS-treatment N = 36, FS-retreatment N = 18; ^d^ FS-treatment N = 39, FS-retreatment N = 20; PCOS = polycystic ovary syndrome.

**Table 2:** Ongoing treatment at relapse and treatment of relapse; statistics for the groups hysterectomy and FS-retreatment.

			Group, N (%) ^a^		
		All Patients N = 26	Hysterectomy N = 6	FS-Retreatment N = 20	*p*-Value ^b^
**Ongoing treatment at relapse**	No Therapy	7 (26.9)	1 (16.7)	6 (30.0)	
	IUD	7 (26.9)	2 (33.3)	5 (25.0)	
	IUD + Met	3 (11.5)	2 (33.3)	1 (5.0)	
	Cyclic Progesterone	8 (30.8)	1 (16.7)	7 (35.0)	
	Continuous Progestin	1 (3.9)	0	1 (5.0)	0.46
**Treatment of relapse**	Hysterectomy	6 (23.1)	6 (100)	0	
	IUD + GnRha	7 (26.9)	0	7 (35.0)	
	IUD + MA+Met	5 (19.2)	0	4 (20.0)	
	IUD + MA	4 (15.4)	0	5 (25.0)	
	Continuous Progestin	4 (15.4)	0	4 (20.0)	NA
**Ovarian stimulation before relapse**		7 (26.9)	2 (33.3)	5 (25.0)	1.00

^a^ Column %; FS = fertility sparing; ^b^ *t*-test for independent variables or Wilcoxon test; NA = not applicable; MA = Megestrol Acetate; Met = Metformin; IUD = Levonorgestrel Intrauterine Device; GnRHa = Gonadotropin-Releasing Hormone Analogue.

**Table 3:** Obstetrical outcomes of primary fertility-sparing treatment and retreatment.

		Group, N (%) ^a^		
	All Patients N = 61	FS-Treatment N = 41	FS-Retreatment N = 20	*p*-Value ^b^
**Try to conceive**	38 (62)	24 (59)	14 (70)	0.83
**Infertility treatment**	21/38 (55)	10/24 (42)	11/14 (79)	0.28
**Post-treatment pregnancy**	29/38 (76)	22/24 (92)	7/14 (50)	0.30
**Live Birth**	19/38 (50)	15/24 (63)	4/14 (29)	0.37
**Miscarriages**	10/29 (34)	7/22 (32)	3/7 (43)	0.70

FS = fertility sparing; ^a^ Column % except for infertility treatment, post-treatment pregnancy, live birth and miscarriages as indicated; ^b^ Fisher’s exact test.

## Data Availability

The data presented in this study are available upon request from the corresponding author. The data are not publicly available due to patients’ privacy restrictions. The data are safely stored in a private database of the European Institute of Oncology, Milan, Italy.
